# Genomic and clinical findings in myeloid neoplasms with *PDGFRB* rearrangement

**DOI:** 10.1007/s00277-021-04712-8

**Published:** 2021-12-02

**Authors:** Danika Di Giacomo, Martina Quintini, Valentina Pierini, Fabrizia Pellanera, Roberta La Starza, Paolo Gorello, Caterina Matteucci, Barbara Crescenzi, Paolo Fabio Fiumara, Marinella Veltroni, Erika Borlenghi, Francesco Albano, Fabio Forghieri, Monica Maccaferri, Francesca Bettelli, Mario Luppi, Antonio Cuneo, Giuseppe Rossi, Cristina Mecucci

**Affiliations:** 1 Department of Medicine and Surgery, Center for Hemato-Oncology Research (C.R.E.O.), University of Perugia, Perugia, Italy; 2 Department of Chemistry, Biology and Biotechnology, University of Perugia, Perugia, Italy; 3 Division of Hematology, AOU Policlinico-San Marco, Catania, Italy; 4 Department of Pediatric Oncology-Hematology, Meyer Children’s Hospital, Florence, Italy; 5 Hematology Unit, ASST-Spedali Civili, Brescia, Italy; 6 Hematology Unit, Department of Emergency and Organ Transplantation, University of Bari, Bari, Italy; 7 Section of Hematology, Department of Medical and Surgical Sciences, University of Modena and Reggio Emilia, AOU Policlinico, Modena, Italy; 8 Hematology, Department of Medical Sciences, St. Anna University Hospital, 44124 Ferrara, Italy

**Keywords:** *PDGFRB*, Chromosome translocations, Imatinib, Molecular monitoring, *KAZN*

## Abstract

Platelet-derived growth factor receptor B (*PDGFRB*) gene rearrangements define a unique subgroup of myeloid and lymphoid neoplasms frequently associated with eosinophilia and characterized by high sensitivity to tyrosine kinase inhibition. To date, various *PDGFRB/*5q32 rearrangements, involving at least 40 fusion partners, have been reported. However, information on genomic and clinical features accompanying rearrangements of *PDGFRB* is still scarce. Here, we characterized a series of 14 cases with a myeloid neoplasm using cytogenetic, single nucleotide polymorphism array, and next-generation sequencing. We identified nine *PDGFRB* translocation partners, including the *KAZN* gene at 1p36.21 as a novel partner in a previously undescribed t(1;5)(p36;q33) chromosome change. In all cases, the *PDGFRB* recombination was the sole cytogenetic abnormality underlying the phenotype. Acquired somatic variants were mainly found in clinically aggressive diseases and involved epigenetic genes (*TET2*, *DNMT3A*, *ASXL1*), transcription factors (*RUNX1* and *CEBPA*), and signaling modulators (*HRAS*). By using both cytogenetic and nested PCR monitoring to evaluate response to imatinib, we found that, in non-AML cases, a low dosage (100–200 mg) is sufficient to induce and maintain longstanding hematological, cytogenetic, and molecular remissions.

**Supplementary Information:**

The online version contains supplementary material available at 10.1007/s00277-021-04712-8.

## Introduction

The *PDGFRB* gene, at 5q32, encodes for the ß chain of the cell surface receptor for platelet-derived growth factor (PDGFRß), a class III receptor tyrosine kinase (RTK) that activates signaling pathways involved in cell growth and differentiation [1]*. PDGFRB* is a frequent target of chromosomal translocations in a subgroup of hematological malignancies recognized in the 2017 World Health Organization (WHO) as a stand-alone category under “Myeloproliferative neoplasms with eosinophilia and gene rearrangement” [2. These disorders are presenting as chronic myeloid neoplasms, frequently as chronic myelomonocytic leukemia with eosinophilia [2], although (hyper)-eosinophilia is not invariably present [3, 4]. Less frequently, *PDGFRB* is rearranged in lymphoid malignancies, including cases of both B- and T-cell acute lymphoblastic leukemia/lymphoma (ALL) [1, 4–6]*.* In addition, in the same patient, a *PDGFRB* rearrangement may underlie the occurrence of two different malignancies of both myeloid and lymphoid lineages, which may be diagnosed concomitantly or sequentially [7, 8]. To date, at least 40 fusion translocation partners of *PDGFRB* have been identified. Among them, *ETV6* is the most frequently involved gene as a consequence of the t(5;12)(q32;p13) [4], while all other partners are rare and often found in single cases [9]. Whatever the partner, the *PDGFRB* gene always participates in the fusion with its 3′ end tyrosine kinase domain, resulting in the constitutive activation and in the deregulation of downstream signaling cascades, including Ras/mitogen-activated protein kinase, phosphatidylinositol 3′-kinase, and phospholipase-5γ pathways [4]. Information on somatic gene variants in cases with a *PDGFRB* rearrangement is still scarce mainly affecting *ASXL1*, *TET2*, *STAG2*, *DNMT3A*, *NRAS*, *ZRSR2*, *BCOR*, and *STAT5B* [4, 7, 10, 11]. Male predominance and a median age at onset in the late 40 s [2] have been established. Most patients have splenomegaly and/or hepatomegaly [2]. Rapid response and long-term remission are obtained with tyrosine kinase inhibitors. In particular, imatinib is the treatment of choice for this group of neoplasms [3, 4, 9]. Although this treatment is successful, few series of cases with clinical and molecular monitoring have been reported [3, 4, 12].

We carried out an in-depth characterization of genomic events accompanying the 5q32 rearrangement in a series of 14 *PDGFRB*-positive cases recruited in our center during the last 23 years. In addition, we evaluated the response to imatinib by cytogenetic and molecular monitoring.

## Materials and methods

### Patients

Patients with a myeloid neoplasm and a rearrangement of *PDGFRB* were recruited from the files of the Laboratory of Cytogenetics at the Department of Medicine and Surgery-Hematology section of the University of Perugia (Table 1). Data collection was done in accordance with the Declaration of Helsinki and its later amendments. The study was approved by the Bioethics Committee University of Perugia (number 2014–0259). All patients or their parents/guardians have provided informed consent for sample collection and use in approved research studies.

**Table 1:** Demographic, clinical, and hematological features of 14 cases with *PDGFRB* rearrangement

Case	Sex/age	Presentation								Clinical and hematological diagnosis	Organomegaly	Lymphadenopathy	LDH (U/L)	Karyotype	Fusion gene	Treatment before imatinib	Response	Previously reported
		WBC (× 10^9^/L)	HGB (g/dL)	MCV (fL)	PLT (× 10^9^/L)		Eosinophils (× 10^9^/L)	Monocytes (× 10^9^/L)										
Chronic presentations																		
1	M/41	85.4	11.1	68.3	183		6.5	2.2		MDS/MPN-U	Splenomegaly Hepatomegaly	Superficial	364	46,XY,t(5;12)(q33;p13)[17]	*ETV6::PDGFRB*	Hydroxyurea	No	No
2	M/68	57.2	12.9	91	240		3.4	1.7		MDS/MPN-U	Hepatomegaly	No	695	46,XY,t(5;12)(q33;p13)[16]/46,XY[4]	*ETV6::PDGFRB*	Steroids and hydroxyurea (3 months)	No	No
3	M/45	40.3	14.3	n.a	187		3.2	2		CMML	Splenomegaly	n.a	n.a	46,XY,add(1)(p36),del(5)(q33q35), der(12)del(12)(p13)add(12)(q22 ~ q24) [18]. ish t(1;12;5;12)(WCP1 + ,WCP12 + ;WCP12 + ,WCP1 + ;WCP5 + ,WCP12 + ;WCP12 + , WCP5 +)/46,XY[2]	*ETV6::PDGFRB*	α-IFN (1 month)	No	Yes [20]
4	M/26	60.8	7.8	90.7	9		1.89	2.91		MDS/MPN-U	Splenomegaly Hepatomegaly	Superficial	581	46,XY,t(5;12)(q31;p13)[10]/46,XY[1]	*ERC1::PDGFRB*	No	-	No
5	M/21	21.6	16.4	n.a	190		8	n.a		CEL	Splenomegaly	No	n.a	46,XY,t(1;5)(q21;q33)[29]/46,XY[1]	*TPM3::PDGFRB*	α-IFN (10 years)	HR	Yes [17, 18]
6	F/31	15.4	14.2	n.a	171		3.2	n.a		CEL	Splenomegaly	n.a	153	46,XX,t(5;14)(q33;q32)	*CCDC88C::PDGFRB*	No	–	Yes [19]
7	F/2	23	12.2	85	346		2.3	1.33		MDS/MPN-U	Splenomegaly	n.a	288	46,XX,t(5;14)(q33;q32)	*CCDC88C::PDGFRB*	Thioguanine (6 months)	No	No
8	F/75	11.2	12.8	100.6	236		2	1.34		MDS/MPN-U	No	n.a	n.a	46,XX,t(5;9)(q33;q34)[20]	*TSC1::PDGFRB*	No	–	No
9	F/35	38.6	13.5	n.a	336		14.4	13.1		CMML	Splenomegaly	n.a	n.a	46,XX,t(5;16)(q33;p13)[11]/46,XX[4]	*NDE1::PDGFRB*	Steroids and hydroxyurea (5 months)	HR	Yes [21, 23]
10	M/49	33	10.3	n.a	398		2	1		aCML	No	No	n.a	46,XY,t(5;10)(q33;q22)[17]/46,XY[1]	*CCDC6::PDGFRB*	Hydroxyurea (18 weeks) -cytarabine + steroids (7 days)-hydroxyurea + thioguanine	No	Yes [16]
11	M/40	16.3	10	90.6	246		2.2	1.11		MDS/MPN-U	Splenomegaly	n.a	n.a	46,XY	*TNIP1::PDGFRB*	No	–	Yes [24]
**Aggressive/unusual presentations**																		
12	M/66	12.6	13.7	91	256	n.a		n.a		Myeloid sarcoma	No	Superficial	Normal	46,XY,t(5;12)(q33;p13)[14]/46,XY[3]	*ETV6::PDGFRB*	nilg aml 06.02 (5 months)	HR, CCR, CMR	No
13	M/36	3.1	11.4	n.a	97	n.a		0.34		Relapsed-AML*	Splenomegaly Hepatomegaly	n.a	n.a	46,XY,t(5;12)(q33;p13.3)[9]/92,XXYY ,t(5;12)(q33;p13.3)X2[12]	*ERC1::PDGFRB*	No	–	Yes [22]
14	M/33	106.8	7.9	111.3	43	1.1		12.8		AML	Splenomegaly	No	1604	46,XY,t(1;5)(p35;q33)	*KAZN::PDGFRB*	No	–	No

*MDS/MPN-U* myelodysplastic/myeloproliferative neoplasm-unclassifiable, *CMML* chronic myelomonocytic leukemia, *AML* acute myeloid leukemia, *CEL* chronic eosinophilia leukemia, *aCML* atypical chronic myeloid leukemia, *WBC* white blood cells, *HGB* hemoglobin, *MCV* mean corpuscular volume, *PLT* platelet, *HR* hematological remission, *CCR* complete cytogenetic remission, *CMR* complete molecular remission, *n.a.* not available

^*^The previous diagnosis was of AML M5a 4 years before, followed by auto-HSCT [22]

### FISH

Fluorescence in situ hybridization (FISH) was used to investigate *PDGFRB* involvement in our cohort, to identify translocation partners, and to assess cytogenetic response. A complete list of the genomic probes is reported in Supplementary Table 1. *PDGFRB* was studied by using either a commercial break-apart probe (LSI PDGFRB Break Apart, Abbott Molecular Diagnostics, Rome, Italy) or home-brew FISH assays with bac probes (RP11-759G10, 149,562,887–149,746,958, for the 5′, and RP11-100O5, 149,274,400–149,455,385, for the 3′) and even more specific cosmid clones: cosmid 4–1, for the 5′, and 9–4, for the 3′ of the gene [13]. The 1p36/*KAZN* breakpoint was characterized using a series of locus-specific probes mapping at 1p36–1p35 regions (Supplementary Table 1). Genomic coordinates are referred to GRCh37/hg19 assembly. One to two hundred nuclei and at least 7 abnormal metaphases were analyzed in each experiment. Follow-up analyses were carried out in one thousand nuclei. Cutoffs were assumed at the upper limit value obtained in 500 cells from a normal donor. FISH was used to assess the cytogenetic response in 13/14 cases (nos. 1–9, 11–14) with a monitoring range of 1–12 months.

### Single nucleotide polymorphism array (SNPa)

Copy number variations (CNVs), gains, losses, and copy neutral loss of heterozygosity (cnLOH) were studied by SNP array in 9 cases with available material at diagnosis (nos. 1–4, 7–8, 11, 13–14) using a Cytoscan HD Array Kit (Affymetrix/Thermo Fisher Scientific, Santa Clara, CA, USA) following manufacturer’s instructions. Data were analyzed by Chromosome Analysis Suite (ChAS 4.1) software with hg19 build as reference. Filters were set at 400 kb, 50 markers for CNVs, and 10 Mb, 50 markers for cnLOH. Polymorphic copy number variants were excluded from the analysis by comparing with the Database of Genomic Variants (http://dgv.tcag.ca/dgv/app/home).

### RT-PCR

Total RNA was extracted by Trizol reagent (Invitrogen, Carlsbad, CA, USA), according to the manufacturer’s protocol. One microgram was retrotranscribed using 50 U of SuperscriptII (Invitrogen). Nested PCR was performed to identify the fusion transcripts in 13/14 cases (nos. 1–9, 11–14) and for monitoring the minimal residual disease in 11 cases (nos. 1–4, 7–9, 11–14) with a monitoring range of 1–12 months (primer details are reported in Supplementary Table 2).

### Treatment

Therapeutic regimens and responses for all patients are reported in Table 1 and .Table 2 Cytoreductive treatment was administered in eight cases (nos. 1–3, 5, 7, 9–11; Table 1) before the cytogenetic finding of a *PDGFRB* involvement was available. Thereafter, imatinib was administered as monotherapy in 13/14 cases (Table 2). Seven cases (nos. 1–2, 4, 6–8, 11) received a low dosage (100–200 mg/die), whereas 4 cases (nos. 3, 9, 13–14) received a high dosage (400 mg/die). In 2 cases (nos. 3 and 9), the dosage was reduced during follow-up. In 2 cases (nos. 5 and 12), the dosage was not available.

**Table 2:** Imatinib regimens and monitoring after treatment

Case	Imatinib dosage at diagnosis	Imatinib dosage at follow-up	CCR (months from imatinib)	CMR (months from imatinib)	Relapse	Last follow-up (months after imatinib)
1	100 mg	200 mg	+ 24	+ 24	No	+ 30^a^
2	100 mg	100 mg	+ 4	+ 18	No	+ 30^a^
3	400 mg	200 mg	+ 1	+ 25	No	+ 222^a^
4	100 mg	100 mg	+ 9	+ 9	No	+ 61^a^
5	n.a	n.a	+ 11	n.a	n.a	+ 33*
6	100 mg	200 mg	+ 24	n.a	No	+ 155*
7	100 mg	200 mg	+ 8	+ 32	No	+ 109^a^
8	100 mg	200 mg	+ 40^§^	+ 40^§^	No	+ 42^a^
9	400 mg	100 mg	+ 12	+ 44	No	+ 129^†^
11	100 mg	200 mg	+ 12	No	No	+ 72^a^
12	n.a	n.a	Maintained after CHT	Maintained after CHT	B-ALL	+ 11^†^
13	400 mg	400 mg	+ 4	No	No	+ 9^†^
14	400 mg	400 mg	+ 7	+ 10^#^	AML	+ 36^†^

*CCR* complete cytogenetic remission, *CMR* complete molecular remission, *CHT* chemotherapy, *B-ALL* B cell acute lymphoblastic leukemia, *n.a.* not available

^a^Alive

^§^First evaluation after treatment

^#^Obtained after 8 months of imatinib therapy followed by 2 months of induction chemotherapy

^†^Death

*Lost at follow up

### Targeted NGS

Mutational analysis of thirty genes mutated in myeloid malignancies and included in the Myeloid Solution SOPHiA GENETICS (Saint‐Sulpice, Switzerland) was performed following the manufacturer’s instructions on 13 cases at diagnosis (patient nos. 1–4, 6–14), in 8 cases (nos. 1, 4, 7–8, 11–14) also at cytogenetic and/or molecular remission, and in one case (no. 14) also after HSCT. The resulting captured libraries were further processed on a Miseq® sequencing platform (Illumina, San Diego, CA, USA). FASTQ sequencing files were uploaded to SOPHiA DDM® platform version 4 and, following adapter trimming and quality filtering, reads were aligned to the human reference genome (hg19 assembly) through the artificial SOPHiA™ intelligence. Variant calling of the resulted alignments was then performed using an in-house somatic variant caller, which takes into account the background noise level at each region. Only exonic and splice sites variants with MAF < 0.01 were taken into consideration and classified according to ACMG criteria [14] using the Varsome database [15]. Mutations with a variant allele frequency (VAF) of 40–60% or > 90% and/or associated with a clinical phenotype reported in OMIM (Online Mendelian Inheritance in Man, https://omim.org/) were defined as germline. All other variants were defined as somatic.

## Results

Fourteen cases were collected. The median age at diagnosis was 38 years (range: 2–75) with a male/female ratio of 2.5 (Table 1). Eleven of fourteen patients were first observed in a non-aggressive phase (6 MPN/MDS, 2 CEL, 2 CMML, 1 aCML), 2 cases were first diagnosed as acute myeloid leukemia, and 1 case was diagnosed as myeloid sarcoma (Table 1). Thirteen of fourteen cases (93%) presented with leukocytosis (range: 11.2–106.8 × 10^9^/L); eosinophilia (≥ 0.5 × 10^9^/L) or hyper-eosinophilia (≥ 1.5 × 10^9^/L) was demonstrated in 12 of them (range: 1.07–14.4 × 10^9^/L) (Table 1). Six cases showed monocytosis (≥ 1.5 × 10^9^/L). Anemia was seen in seven cases and thrombocytopenia in three. Hepatomegaly and/or splenomegaly was present in eleven cases (Table 1), and lymphadenopathy was noticed in three (Table 1). The serum lactate dehydrogenase level was available in seven cases and resulted increased in five (288–1604 U/L). Skin lesions have been noticed at diagnosis in two cases (nos. 7 and 9, Table 1).

### Cytogenetics and molecular findings

**Fig. 1:**
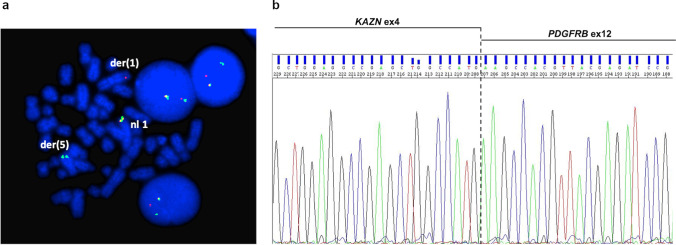
Cytogenetic and molecular characterization of the novel *KAZN::PDGFRB* fusion. **a** FISH break-apart assay with fosmids WI2-0883F15 (spectrum green) and WI2-0968K05 (spectrum orange) for *KAZN*/1p36.21 showed a fusion signal on normal chromosome 1, a red signal on der(1), and a green signal on der(5) in case no. 14 (Table 1). **b** Direct sequencing showed an in-frame fusion joining exon 4 of *KAZN* to exon 12 of *PDGFRB*. GenBank accession numbers: NM_201628.3 for *KAZN* and NM_002609.3 for *PDGFRB*. nl, normal; ex, exon

Involvement of 5q32 was detected by conventional cytogenetics in 13/14 patients while it was cryptic in case no. 11 (Table 1). In case no. 13, with t(5;12), hyperdiploidy was predominant. FISH revealed *PDGFRB* involvement and identified the translocation partner in all cases. Seven partners were already reported [16–24]. RT-PCR confirmed the presence of the corresponding fusion transcript in all cases with available material (12/14; nos. 1–4, 6–9, 11–14). Nine different partner genes were found (Table 1). Among them, *ETV6* (*n* = 4), *ERC1* (*n* = 2), and *CCDC88C* (*n* = 2) were recurrent partners. Moreover, in case no. 14, we identified *KAZN* at 1p36.21 as a novel partner gene of *PDGFRB*. Metaphase FISH identified the 1p breakpoint between RP11-1079F4, flanking the 5′, and RP11-317H5, covering the 3′ of the gene. Additional experiments, showing the splitting of WI2-0883F15 and WI2-0968K05 fosmids, narrowed the breakpoint between exons 4 and 5 of *KAZN* (Fig. 1a). RT-PCR confirmed an in-frame fusion between exon 4 of *KAZN* (NM_201628.3) and exon 12 of *PDGFRB* (NM_002609.3) (Fig. 1b)

### SNParray

SNPa revealed a total of 13 events (3 gains, 8 losses, and 2 copy neutral LOH, Supplementary Table 3) in nine investigated cases. However, they were mainly concentrated in case no. 13 showing nine imbalances (gains at 3q and 8q, and losses at 1p, 2q, 4q, and 5q; Supplementary Table 3). Four events were distributed in case nos. 1, 3, 11, and 14 (Supplementary Table 3). The remaining four cases (nos. 2, 4, 7, and 8) were normal (Supplementary Table 3).

### Mutational analysis

**Fig. 2:**
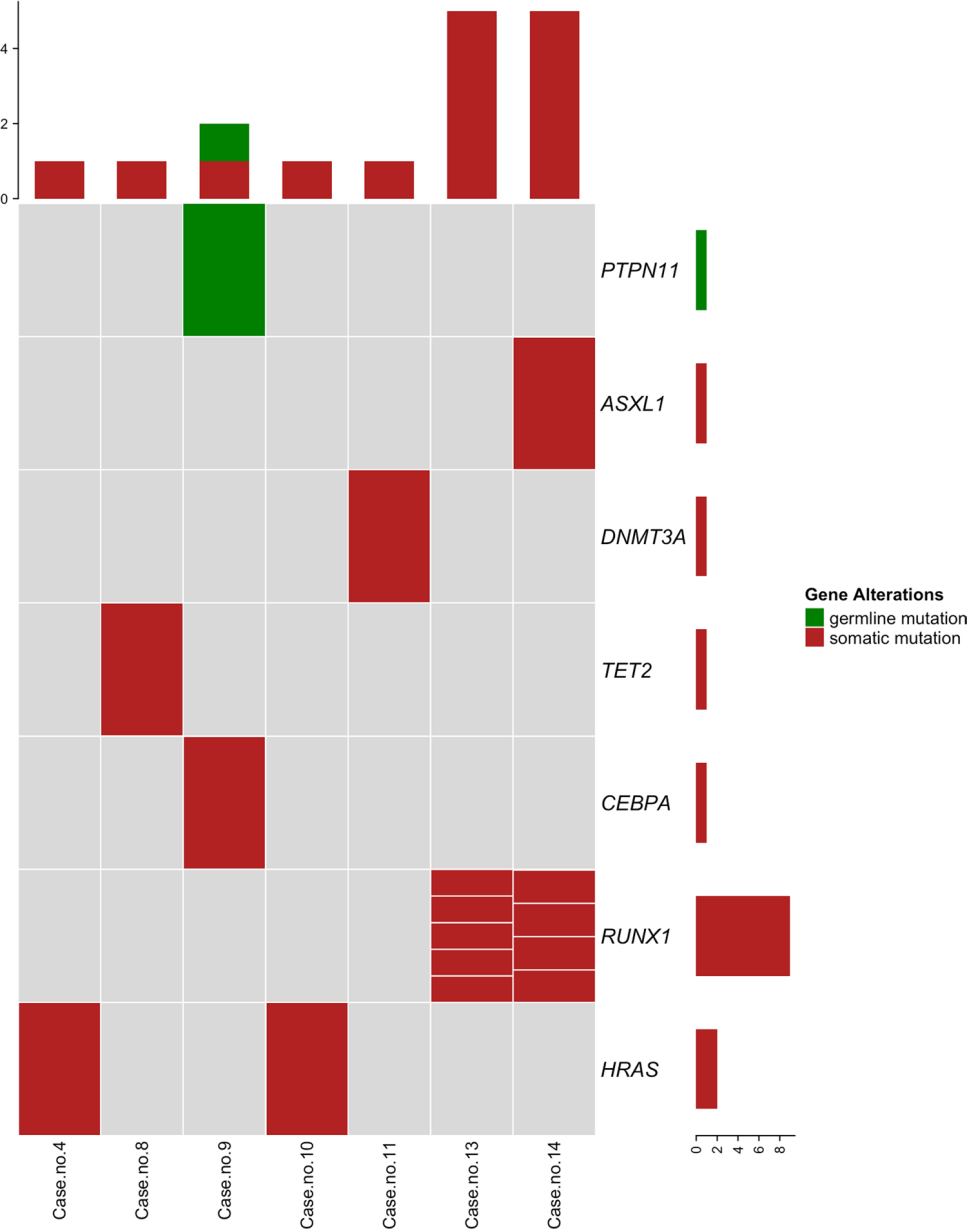
The mutational background of *PDGFRB*-positive cases. Oncoprint heatmap showing mutations found at diagnosis in cases with a rearrangement of *PDGFRB*. Somatic mutations are reported in red and germline mutations in green; gray, non-mutated genes

NGS was applied in 13 cases at diagnosis. Seven of them showed a total of 16 variants (range: 0–5 per case) (Fig. 2 and Table 3). One germline mutation at *PTPN11* was confirmed in patient no. 9 (Fig. 2 and Table 3). A total of five somatic variants affecting *HRAS* (*n* = 2), *TET2*, *DNMT3A*, and *CEBPA* (*n* = 1 each) were found in 5 out of the 10 cases with chronic presentation (nos. 4, 8–11; 1 mutation/case). Longitudinal studies showed disappearance of *HRAS* and *TET2* variants at remission in case nos. 4 and 8, respectively, whereas *DNMT3A* mutation load increased at cytogenetic remission in case no. 11 (Table 3). In the two cases with AML at presentation (nos. 13 and 14), we found a total of 10 somatic mutations (5 mutation/case) (Fig. 2 and Table 3). In both cases, *RUNX1* was affected by multiple variants (*n* = 9), while one mutation at *ASXL1* was found in case no. 14 (see Fig. 2, Table 3, and below for details).

**Table 3:** Sequence variants identified in *PDGFRB*-positive cases and their longitudinal monitoring

	**Case no 4**		**Case no 8**		**Case no 9**	**Case no 10**	**Case no 11**		**Case no 13**		**Case no 14**		
	Diagnosis	CCR/CMR	Diagnosis	CCR/CMR	Diagnosis	Diagnosis	Diagnosis	CCR	Diagnosis	CCR	Diagnosis	CCR	Post-HSCT
***FLT3***											Absent	c.1804_1805ins120, p.Leu601_Lys602ins40 (2.6%)	Absent
***PTPN11***					c.178 G > T, p.Gly60Cys (43.3%)								
***ASXL1***											c.2277C > A, p.Cys759* (45.4%)	c.2277C > A, p.Cys759* (42.7%)	c.2277C > A, p.Cys759* (1.4%)
***DNMT3A***							c.2645G > A, p.Arg882His (8.5%)	c.2645G > A, p.Arg882His (25.9%)					
***TET2***			c.1123G > T, p.Glu375* (29.7%)	Absent									
***CEBPA***					c.646A > G, p.Thr216Ala (2.5%)								
***RUNX1***									c.500G > T, p.Ser167Ile (17.8%)	Absent	c.777dupT, p.Asn260* (41.6%)	c.777dupT, p.Asn260* (16%)	Absent
									c.586A > C, p.Thr196Pro (17.1%)	Absent	c.497G > A, p.Arg166Gln (1.3%)	c.497G > A, p.Arg166Gln (4.8%)	c.497G > A, p.Arg166Gln (1.5%)
									c.496C > T, p.Arg166* (16.7%)	Absent	c.472_473insGG, p.Phe158Trpfs*19 (1.9%)	c.472_473insGG, p.Phe158Trpfs*19 (13.3%)	Absent
									c.1011del, p.Ala338Argfs*256 (7.3%)	Absent	c.941_942insCT, p.Ala315Leufs*14 (0.7%)	c.941_942insCT, p.Ala315Leufs*14 (2.2%)	Absent
									c.424_445dup, p.Ala149Glyfs*2 (6.8%)	Absent			
***HRAS***	c.404G > A, p.Arg135Gln (7.1%)	Absent				c.412G > T, p.Gly138Cys (2.8%)							

Variance allele frequency (VAF) is indicated in brackets

*CCR* complete cytogenetic remission of the *PDGFRB* rearrangement, *CMR* complete molecular remission of the *PDGFRB* rearrangement, *HSCT* hematopoietic stem cell transplant

### Aggressive and unusual presentations

Two cases of our cohort presented as acute myeloid leukemia (nos. 13 and 14) and one case as myeloid sarcoma (no. 12).

### Case no. 12

A 66-year-old man was diagnosed with myeloid sarcoma in another center based on a lymph node biopsy. At the same time, we found t(5;12)/*PDGFRB::ETV6* and the absence of somatic mutations in bone marrow cells. Hematological, cytogenetic, and molecular response were achieved after 2 cycles of chemotherapy [25]. Remission was maintained by imatinib which was discontinued after 5 months because of iatrogenic interstitial infiltrates and pulmonary fibrosis. Four months later, the patient developed a B-ALL but material was not available for our cytogenetic-molecular studies. He died in aplasia after chemotherapy.

### Case no. 13

A 36-year-old man received imatinib only at relapse, occurring 4 years after the first identification of a t(5;12)-positive AML that underwent chemotherapy and autologous transplant. At relapse, in addition to the *PDGFRB* translocation, we found five somatic variants at the *RUNX1* gene (Fig. 2 and Table 3). The patient was treated with imatinib (400 mg/day) followed by chemotherapy. The disappearance of hematological and cytogenetic remissions and *RUNX1* mutations was observed, followed by an unrelated HSCT. The patient died because of transplant-related events.

### Case no. 14

A 33-year-old man was first observed because of marked splenomegaly and leukocytosis (Table 1) with 22% of circulating myeloblasts and promonocytes, and bone marrow blasts < 20%. A t(1;5) translocation plus four somatic variants at *RUNX1* and one at *ASXL1* (Fig. 2 and Table 3) was documented. Cytogenetic remission was observed after 6 months of imatinib at 400 mg/die although increased blast cells (20–30%) were found in the bone marrow aspirate. At that time, a complex mutational landscape was identified. The *ASXL1-*positive cells persisted with unchanged VAF, while involvement of *RUNX1* showed a decrease of the non-sense variant c.777dupT, p.Asn260*, but an increase of the three additional variants already found at diagnosis (Table 3). Moreover, a *FLT3-*ITD was identified in a small-size cell population. Treatment was standard chemotherapy and subsequent HSCT from an HLA-identical sibling donor. AML relapsed 1 year after HSCT, when bone marrow cells were negative for both *PDGFRB* and *RUNX1* c.777dupT, p.Asn260*, but still positive for *ASXL1* and for 1 out of the 3 *RUNX1* variants identified at diagnosis (c.497G > A, p.Arg166Gln) (Table 3). Unfortunately, massive splenomegaly and CNS involvement occurred, requiring palliative splenic and cranio-spinal irradiation. The patient died of progressive disease 24 months after HSCT.

### Imatinib dosage

In 10/11 cases first observed with a non-aggressive disease phase, imatinib was administered as sole treatment. Six cases (nos. 1–3, 5, 7, and 9) received a cytoreductive therapy before imatinib while four cases (nos. 4, 6, 8, and 11) received imatinib as front-line therapy (Table 1). In nine cases, precise information on dosage, response, and follow-up was available. A dosage of 100 mg/die induced complete cytogenetic and molecular remission in three cases (nos. 2, 4, and 7) while an increase to 200 mg/die was necessary in four other cases (nos. 1, 6, 8, and 11). In two cases (nos. 3 and 9), a higher dosage (400 mg/die) induced cytogenetic and molecular remission that was maintained by a dosage of 200 mg in case no. 3 and 100 mg in case no. 9. Case no. 3, still in monitoring, has the longest follow-up of 18 years, while case no. 9 died of sepsis of unknown origin in disease remission at + 129 months. A long-standing history was observed in our pediatric case (no. 7) who was started on a dosage of 100 mg/die and obtained a molecular remission after 32 months. However, after an additional 19 months, we detected molecular relapse and imatinib was increased to 200 mg/die, obtaining a second molecular remission after 10 months. The disease is stable with negative cytogenetic and molecular tests at + 44 months from the second remission and at + 105 months from the first imatinib administration. The median survival from the start of imatinib treatment in the nine chronic cases is 66.5 months (range: 30–222 months). Eight cases are still in follow-up.

### Monitoring of PDGFRB rearrangements

Both FISH and nested or semi-nested RT-PCR were used for disease monitoring during imatinib treatment. FISH documented complete cytogenetic remission in all 13 cases with a median of 9 months after start of treatment. Molecular remission was also documented in 9 of 11 tested cases with a median of 24 months after start of treatment. In case no. 13, positivity disappeared only after HSCT, while case no. 11, who discontinued imatinib for 10 weeks due to kidney transplantation, was positive at last follow-up (+ 72 months from imatinib administration).

In four cases with complete monitoring (nos. 2–3, 7, and 9), the median time lapse between cytogenetic and molecular remission was 24 months (range: 14–32). The earliest cytogenetic remission was obtained in case no. 3 after 1 month of treatment, although the disease persisted at molecular level up to month 25. In case nos. 1 and 4, remission was simultaneously documented by FISH and RT-PCR after 24 and 9 months of treatment, respectively. The median overall survival after molecular remission in nine cases was 42 months (range: 6–197), with ongoing follow-up in seven cases.

## Discussion

Chromosomal rearrangements involving *PDGFRB* in myeloid malignancies are rare events. Here, we characterized a series of 14 cases with a myeloid neoplasm at diagnosis and a rearrangement of *PDGFRB*, providing data from in-depth genomic characterization by SNPa and NGS analysis. At cytogenetics, the 5q32/*PDGFRB* rearrangement involved multiple partners identified by banding in all cases but one with a cryptic change. Notably, in one case, complex chromosome rearrangements corresponded to a *PDGFRB::ETV6* fusion underlying a four-break translocation. These results confirm the clinical relevance of FISH studies for *PDGFRB* gene rearrangements in patients with myeloid neoplasms, especially when associated with eosinophilia, even in the absence of karyotypic abnormalities. Notably, the 5q32/*PDGFRB* rearrangement was the sole cytogenetic abnormality in our cohort and SNPa confirmed a low burden of co-occurring abnormalities in all cases with chronic disease, strongly supporting the driver role of PDGFRß tyrosine kinase activation to address the clinical phenotype.

This study first identified the *KAZN* gene at 1p36 as a novel partner of *PDGFRB* in one case with a t(1;5)(p36;q33). The *KAZN* gene is involved in intercellular adhesion, cell differentiation, signal transduction, cytoskeletal organization, and apoptosis [26]. To date, *PTBP2*, *TMEM51*, and *MTOR* have been found as fusion partners of *KAZN* by RNA sequencing studies in solid tumors [27, 28], while, as far as we know, rearrangements of *KAZN* have never been described in hematological malignancies. Recurrent *KAZN* variants have not been specifically associated with human tumors (COSMIC, Catalogue of Somatic Mutation in Cancer [29]).

In a literature review, we found only 8 cases in chronic phase with information on the mutational background that showed variants at *TET2*, *ASXL1*, and *STAG2* genes [4, 7, 10]. In our cases presenting with a chronic clinical phenotype, we found a low burden of somatic mutations involving *TET2*, *DNMT3A*, *HRAS*, and *CEBPA*. Notably, five and four variants affected the *RUNX1* gene in our AML case no. 13 and no. 14, respectively. These findings suggest that instability at *RUNX1* is a nonrandom event accompanying an acute clinical phenotype in *PDGFRB* + malignancies. This hypothesis is supported by the work of Stengel et al. [30], who rarely (< 1%) found more than three variants of the *RUNX1* gene in a large series of mutated de novo AML cases. With respect to the nature of *RUNX1* mutations in our acute cases, they are all predicted to be pathogenic. Six of them fell in the Runt-Homology DNA binding Domain (RHD), one fell in the Trans-Activation Domain (TAD), and two non-sense mutations truncated the protein between RHD and TAD.

Longitudinal studies helped us to characterize the behavior of somatic mutations over the disease course. In particular, both *HRAS* and *TET2* gene variants disappeared concomitantly to cytogenetic and molecular remission of the *PDGFRB* abnormality. These cases, in addition to one case with somatic mutation at the *BCOR* gene that disappeared at remission of the *PDGFRB* rearrangement [11], suggest that all these acquired mutations were present in the *PDGFRB* + clonal population sensitive to imatinib. Instead, a peculiar dynamics of somatic gene variants, as compared to the *PDGFRB* rearrangement, was seen in our case no. 14, in which one of the *RUNX1* variants (c.777dupT, p.Asn260*) decreased concomitantly to cytogenetic remission, whereas both the *ASXL1* and the c.497G > A *RUNX1* variants appeared as the leukemic *fil rouge* over the whole disease course in this case, from diagnosis till post-transplant relapse, when *PDGFRB* was absent. Altogether, these results raise the question whether AML in the last case was a second malignancy instead of an acute phase originated by linear clonal evolution [31] of the *PDGFRB-*positive cells. Single-cell analysis will be helpful to better understand the origin of AML in *PDGFRB*-positive cases. Finally, the increased clonal size of *DNMT3A* in another responder (case no. 11) with cytogenetic disappearance of the *PDGFRB* rearrangement is consistent with clonal hematopoiesis, similar to that observed in other myeloid malignancies [32].

Our study confirmed that prognosis in cases with *PDGFRB* rearrangement is deeply influenced by the clinical presentation [3], as our cases first seen with acute disease had a more unfavorable outcome compared to cases with less aggressive hematological phenotypes at diagnosis.

A consensus on the posology of the TKI inhibitor in *PDGFRB*-related disorders presenting in chronic phase is still not available. The present series underlines the low dosage of 100–200 mg as a successful approach to inducing and maintaining cytogenetic and molecular remission. Moreover, molecular analysis is essential to carefully monitoring response to therapy, to evaluate the amount of residual disease, and to address changes of imatinib dosage over the disease course.

## Supplementary Information

Below is the link to the electronic supplementary material.

Supplementary file1 (XLS 35 KB)

Supplementary file2 (XLS 34 KB)

Supplementary file3 (XLS 37 KB)

## Data Availability

All datasets generated during the current study were deposited in NCBI Gene Expression Omnibus (GEO) under accession number GSE182820 (GSE182785 for SNParray and GSE182817 for Sequencing data).
